# The Interaction Between Long Non-Coding RNAs and Cancer-Associated Fibroblasts in Lung Cancer

**DOI:** 10.3389/fcell.2021.714125

**Published:** 2022-01-11

**Authors:** Wenqi Ti, Jianbo Wang, Yufeng Cheng

**Affiliations:** Department of Radiation Oncology, Qilu Hospital, Cheeloo College of Medicine, Shandong University, Jinan, China

**Keywords:** cancer-associated fibroblasts (CAFs), long non-coding RNAs (lncRNAs), tumor microenvironment, exosomes, lung cancer, targeted therapy

## Abstract

Despite great advances in research and treatment, lung cancer is still one of the most leading causes of cancer-related deaths worldwide. Evidence is mounting that dynamic communication network in the tumor microenvironment (TME) play an integral role in tumor initiation and development. Cancer-associated fibroblasts (CAFs), which promote tumor growth and metastasis, are the most important stroma component in the tumor microenvironment. Consequently, in-depth identification of relevant molecular mechanisms and biomarkers related to CAFs will increase understanding of tumor development process, which is of great significance for precise treatment of lung cancer. With the development of sequencing technologies such as microarray and next-generation sequencing, lncRNAs without protein-coding ability have been found to act as communicators between tumor cells and CAFs. LncRNAs participate in the activation of normal fibroblasts (NFs) to CAFs. Moreover, activated CAFs can influence the gene expression and secretion characteristics of cells through lncRNAs, enhancing the malignant biological process in tumor cells. In addition, lncRNA-loaded exosomes are considered to be another important form of crosstalk between tumor cells and CAFs. In this review, we focus on the interaction between tumor cells and CAFs mediated by lncRNAs in the lung cancer microenvironment, and discuss the analysis of biological function and molecular mechanism. Furthermore, it contributes to paving a novel direction for the clinical treatment of lung cancer.

## Introduction

According to global statistics, lung cancer probably accounts for one-fifth of all cancer deaths and is one of the most deadly malignancies (Bray et al., 2020). In recent years, despite a large number of novel discoveries that have been achieved in treatment, the prognosis of patients with advanced lung cancer is still poor due to the delayed diagnosis of the disease, with the 5-year survival rate less than 5% (Hirsch et al., 2017). Therefore, it is an urgent problem to dig into the pathogenesis of lung cancer and improve the therapeutic effect of patients.

Tumor cells do not exist independently, but are surrounded by tumor microenvironment rich in stromal components and vascular networks. Microenvironment-mediated tumor initiation, progression, metastasis, and even drug resistance are the result of continuous interactions between tumor cells and the surrounding stroma. Cancer-associated fibroblasts, a type of permanently activated fibroblasts, are the most prominent stromal components and have been proved to have profound effects on tumor regulation. CAFs are the most dominant cells that synthesize and reshape extracellular matrix (ECM) in the tumor microenvironment, which contribute to increasing the hardness of tumor tissue and thus increase the ability of local invasion and metastasis. Meanwhile, CAFs are also important sources of many growth factors, chemokines, cytokines, such as vascular endothelial growth factor (VEGF), fibroblast growth factor (FGF), platelet-derived growth factor (PDGF), transforming growth factor-β (TGF-β), interleukin-6 (IL-6) and stromal cell-derived factor-1 (SDF-1). CAFs play multiple carcinogenic roles by regulating these factors and participating in various signaling pathways. A better understanding of the contribution of CAFs to tumor progression will lead to promising therapeutic approach interventions for stroma.

Ultimately, tumor is a genetic disease that alters the flow of information in cells to change cell homeostasis and promote its proliferation. The Cancer Genome Atlas (TCGA) and the Cancer LncRNA Census (CLC) have provided cancer researchers with useful datasets and annotated data that accelerate our understanding of molecular level of tumor, including the role of lncRNAs in tumor development (Taniue and Akimitsu, 2021). A growing body of evidence shows that lncRNAs participate in crosstalk between tumor cells and microenvironment. Here, we summarize the relationship between lncRNAs and CAFs, as well as specific role and mechanism of lncRNAs in CAFs’ promotion of lung cancer invasion, immunosuppression and therapeutic resistance, finally explore the diagnostic, prognostic and therapeutic value of lncRNAs in the TME.

## The Current Understanding of Cancer-Associated Fibroblast Biology

Tumor is a complex ecosystem, including not only tumor cells, but also many stromal components. The stroma is made up of many distinct cell types, including fibroblasts, adipocytes, myeloid-derived suppressor cells (MDSCs), macrophages, lymphocytes, smooth muscle, blood vessels, and extracellular matrix (ECM). Together, they compose the microenvironment in which the tumor is located, known as the tumor microenvironment (TME) (Li et al., 2007; Zhang H. et al., 2020). Although the “seed and soil” theory was first proposed in the 1880s, the role of TME in affecting cancer initiation and development has not received widespread concern until recent decades (Paget, 1989; Chen and Song, 2019). There are differences between TME and normal tissue environment in terms of tissue structure, pH level, cell nutritional status, metabolism, and so on. Although TME may have some anticancer actions, it generally provides the best conditions for the growth, invasion and metastasis of various types of tumor cells. It can be said that TME is the essential “soil” for the breeding of tumor “seeds.” Cancer-associated fibroblasts (CAFs), as one of the most important and active components in the tumor microenvironment, regulate tumorigenesis and therapeutic response by synthesizing ECM and secreting varieties of soluble factors (Yeh et al., 2015; Sahai et al., 2020). CAFs isolated from human lung cancer tissue induce EMT and enhance the metastatic potential of cancer cells by activating the IL-6/STAT3 signaling pathway (Wang et al., 2017). Fibroblasts in normal tissues, as the main producers of ECM, are activated during tissue injury, inflammation, and fibrosis, and thus play key roles in tissue repair and regeneration. NFs that are continuously activated in the tumor stroma are referred to CAFs (Chen and Song, 2019; Fang et al., 2020). Therefore, we hypothesized that CAFs could also be used for anticancer therapy in tumors long known as “wounds that do not heal.” Compared to NFs, CAFs have increased proliferation and migration characteristics due to their differential gene and protein expression characteristics (De Wever et al., 2008; Saadi et al., 2010). In addition, CAFs secrete elevated levels of ECM proteins, such as fibronectin and type I collagen, to provide physical scaffolds for tumor tissue (Chan et al., 2017).

### The Origin and Heterogeneity of Cancer-Associated Fibroblasts

There is growing evidence that CAFs are recognized as heterogeneous population of cells. This heterogeneity may be caused by different origins of CAFs (Kalluri, 2016). In-depth research into the origin and activation of CAFs in human malignancies has also expanded our understanding of the phenotypic heterogeneity and functional diversity of CAFs. Although the origin of CAFs has been further clarified by the use of lineage tracing mouse models, it is difficult to draw definitive conclusions about the origin of CAFs when markers of both normal fibroblasts and CAFs are not quite clear (Alcolea and Jones, 2013; LeBleu et al., 2013). Despite several CAFs markers have been identified by immunohistochemistry, including α-smooth muscle actin (α-SMA), fibroblast activation protein-α (FAP-α), vimentin, and fibroblast specific protein-1 (FSP-1) (Kraman et al., 2010). However, the expression of common fibroblast markers is extremely uneven and varies considerably among different CAF subsets. This leads to a limitation in understanding the activation of CAFs. The current consensus is that most CAFs are probably recruited and activated by local tissue fibroblasts, but there are clear examples of other sources (Sahai et al., 2020). CAFs can originate from normal resident tissue fibroblasts. Transforming growth factor-β (TGF-β), fibroblast growth factor 2 (FGF2), epidermal growth factor (EGF), platelet-derived growth factor (PDGF), hypoxia, reactive oxygen species (ROS) and non-coding RNAs are key regulators of fibroblasts activation (Tape et al., 2016; Deng et al., 2020). As a strong inducer of proliferation and fibrosis, TGF-β activates fibroblasts in pancreatic adenocarcinoma (Löhr et al., 2001). Activation of hepatic stellate cells (HSCs) by PDGF results in myofibroid phenotypes, including features such as α-SMA expression, that are transformed into CAFs (Yin et al., 2013). Hypoxia induces epigenetic reprogramming of normal breast fibroblasts, resulting in the pro-glycolytic phenotype of CAFs (Becker et al., 2020).

In addition, other cell types in TME (e.g., epithelial cells, endothelial cells, adipocytes and pericyte) may also participate in CAFs’ differentiation. In breast cancer, kidney cancer, lung cancer and liver cancer, epithelial cells and endothelial cells adjacent to the cancer cells can differentiate into CAFs through epithelial-mesenchymal transition (EMT) and endothelial-mesenchymal transition (EndMT) (Kalluri and Neilson, 2003; LeBleu and Kalluri, 2018). Although conversion of adipocytes to CAFs is not a universal phenomenon in tumors, it has been partially reported in breast cancer. Human adipose tissue-derived stem cells (hASCs) adjacent to breast cancer cells are also one of the sources of CAFs and play an important role in tumor aggression (Jotzu et al., 2011). Evidence of pericyte transformation of CAF is relatively rare (Dulauroy et al., 2012; Bartoschek et al., 2018). Pericytes can be transferred into CAFs in a PDGF—dependent manner (Hosaka et al., 2016). In recent years there has been evidence that cancer stem cells (CSCs), which are thought to be the origin of cancer, can also differentiate into CAFs. It provides a new dimension for CAFs heterogeneity (Osman et al., 2020).

Distant cells outside the TME can also be transformed into CAFs. When mesenchymal stem cells (MSCs) are recruited by colorectal carcinoma microenvrionment, CXCR4/TGF-β1 signaling signaling pathway in this environment can mediate MSCs differentiation into CAFs (Tan et al., 2020). Cancer progression requires stromal support to maintain tumor growth. CAFs, as the main producer of ECM and paracrine signals, play key roles in tumorigenesis, angiogenesis, metastasis, tumor stem cell maintenance and metabolic reprogramming, immunosuppression, and drug resistance. Hence, it’s key to reprogram normal fibroblasts into tumorigenic CAFs.

### Cancer-Associated Fibroblasts: Functional Overview in Lung Cancer

Due to their heterogeneity, CAFs have been shown to promote lung cancer development through a variety of unique mechanisms. CAFs were first shown to promote tumor progression in a prostate cancer model (Olumi et al., 1999). Navab et al. (2016) found that CAFs can enhance the interstitial collagen hardness through overexpression of integrin α11β1, thereby promoting tumor progression in NSCLC. It is known that CAFs are large producers of IL-6 in the cancer microenvironment, inducing EMT through the IL-6/STAT3 signaling pathway and enhancing the metastatic potential of lung cancer cells (Wang et al., 2017). CAFs are also involved in maintaining the immunosuppressive and angiogenic environment that promotes tumor growth and evades immune surveillance. For example, CAFs can induce immunosuppression by recruiting immunosuppressive cells to a tumor site. CAFs can also recruit endothelial progenitor cells into carcinomas by secreting stromal cell derived factor 1 (SDF1), thereby stimulating tumor angiogenesis (Orimo et al., 2005). In addition, CAFs mediated drug resistance was observed in lung cancer. For example, IL-6 from CAFs significantly increases TGF-β1-induced EMT in cancer cells, thereby promoting cisplatin resistance in NSCLC (Shintani et al., 2016). Hepatocyte growth factor (HGF) from CAFs activated Met/PI3K/Akt, up-regulated the expression of GRP78, promoting the resistance of A549 cells to paclitaxel (Ying et al., 2015). Many of these aspects have been reviewed in the past. We pay particular attention to lncRNAs as relatively new biomolecules participated in the interactions with CAFs. Through lncRNAs, the metabolic characteristics of NFs were changed, leading to the activation of CAFs. Activated CAFs enhance malignant biological processes in tumor cells by interacting with lncRNAs, which can promote tumor progression more effectively. LncRNAs also participate in bidirectional communication between tumor cells and CAFs via exosomes, resulting in both types of cells being reprogrammed to maintain malignancy.

## Long Non-Coding RNA: Classification and Functional Characteristics

Based on the Encyclopedia of DNA Elements (ENCODE) project, 93% of the genome is transcribed into RNA, of which only 2% is translated to protein. Those RNAs that lack the ability of coding proteins are known as non-coding RNAs (ncRNAs). There are many types of non-coding RNAs, which can be divided by size into short non-coding RNAs with length less than 200 bp and long non-coding RNAs with more than 200 bp (Brosnan and Voinnet, 2009; Clark et al., 2011; Iyer et al., 2015). Initially, it was believed that lncRNA was a by-product of RNA polymerase II transcription, which was the result of transcriptional junk in the process of genome evolution and had no biological function (Kopp and Mendell, 2018). The development of whole genome and transcriptome sequencing technology has allowed in-depth examination of the noncoding genome. In recent years, the functional roles of lncRNAs have become clearer than initially anticipated.

**FIGURE 1:**
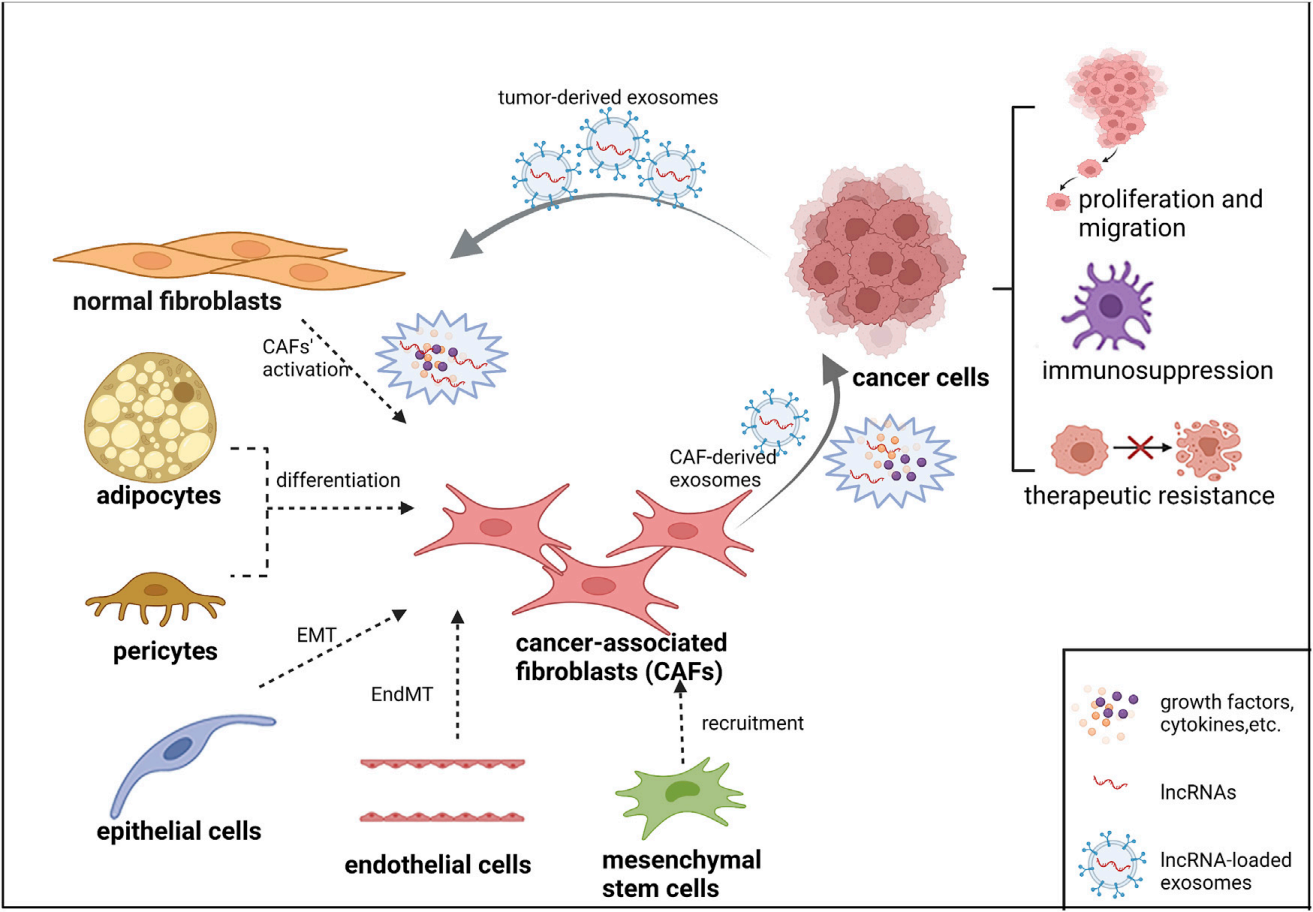
The heterogeneous origin of CAFs and their interactions with tumor cells via lncRNAs.

Multiple mechanisms have been involved in the lncRNA-mediated gene regulation in many diseases, including tumors. This is probably due to their interactions with DNA, RNA or protein at three levels of transcriptional regulation, post-transcriptional regulation and epigenetic regulation. Most studies have demonstrated that the ability of lncRNAs to interact with different biomolecules extensively is of great significance in tumor development, such as tumor proliferation, metabolism, differentiation, apoptosis, migration and drug resistance. A recent series of experimental evidence indicates that their roles in TME are being recognized (Fang et al., 2020). In addition, tumor cells and CAFs can communicate more directly through lncRNA-loaded exosomes. Exosomes are a class of extracellular vesicles (evs) with a diameter of 30–100 nm, carrying microRNAs, lncRNAs, proteins, metabolites, and other bioactive substances. Exosomes secreted by CAFs can influence tumor progression. While exosomes released by cancer cells can also promote the transformation and activation of CAFs. Although the study of lncRNAs in TME has just begun, it can be predicted that significant progress can be made in the study of lncRNAs interaction with CAFs. The heterogeneous origin of CAFs and their interactions with tumor cells via lncRNAs are illustrated in Figure 1. Figure created with BioRender (https://biorender.com).

Normal fibroblasts mainly transformed into CAFs through coordination of cytokines, growth factors, lncRNAs, etc. Epithelial cells, endothelial cells, adipocytes, pericytes and mesenchymal stem cells may also participate in the differentiation of CAFs through different mechanisms. CAFs interact with lncRNAs to maintain tumor proliferation, migration, immunosuppression and therapeutic resistance. LncRNA-loaded exosomes are also widely involved in crosstalk between tumor cells and CAFs.

## The Dynamic Crosstalk Between Tumor Cells and Cancer-Associated Fibroblasts Mediated by Long Non-Coding RNAs

The crosstalk between tumor cells and the tumor microenvironment is deemed to be necessary for tumor progression (Gascard and Tlsty, 2016). CAFs are major participants in TME, promoting tumor progression and metastasis through communication with adjacent tumor cells directly or indirectly. Now accumulating evidence has revealed that lncRNAs, as relatively novel regulatory factors, play critical roles not only in tumor cells but also in the tumor microenvironment. The dysregulation of lncRNAs and exosomal lncRNAs are involved in the dynamic crosstalk between CAFs and tumor cells. By exploring the relationship between lncRNAs, CAFs and cancer cells, using lncRNAs as biomarkers or targets may be a potential way to predict the prognosis of patients and improve the therapeutic effect of lung cancer in the future (Wang et al., 2018). The following details describe the CAFs-related lncRNAs and their potential mechanisms in lung cancer (Table 1).

**TABLE 1:** CAFs-related lncRNAs and their potential mechanisms in lung cancer.

LncRNA	Expression	Target molecules or pathways	Cell lines	Principal functions	References
PCAT-1	Upregulation	miR-182/miR-217 signaling	H1975, A549	Activate CAFs, Immunosuppression, Chemoresistance	Domvri et al. (2020)
TBILA	Upregulation	S100A7/JAB1	A549	promoting progression	Lu et al. (2018)
ANCR	Downregulaton	TGF-β signaling	NCI-H23, NCI-H522	inhibit migration and invasion	Wang et al. (2018)
Snail-1	Upregulation	Snail1 signaling	A549	promote EMT of epithelial lung cancer cells	You et al. (2019)
HOTAIR	Upregulation	caspase-3/BCL-2 signaling	A549	promote cisplatin resistance	Sun and Chen (2021)
ANRIL	Upregulation	caspase-3/BCL-2 signaling	A549	Promote cisplatin resistance	Zhang et al. (2017)
KCNQ1OT1	Upregulation	G1/S transformation	A549	Radiotherapy resistance	Mao (2019)

### Role in the Formation and Activation of Cancer-Associated Fibroblasts

Compared with resting fibroblasts, hyperactivated fibroblasts (i.e., CAFs) have stronger tumor-promoting ability in NSCLC (Navab et al., 2011). In addition to the well-known miRNAs, some studies have confirmed that lncRNAs are also one of the regulatory factors that contribute to the formation and activation of CAFs. By integrating the gene expression profiles of 32 cancer types and clustering the associated lncRNAs, 16 lncRNA modules were obtained. Twelve lncRNAs in one of these were associated with cancer fibroblasts activation. Differentiation of quiescent fibroblasts into cancer-associated phenotypes was reduced by siRNA knockdown experiment (Walters et al., 2019). Snail1 activates cancer-associated fibroblasts (CAF), which require signals from tumor cells such as TGF-β (Alba-Castellón et al., 2016). It was found that lncRNA FLJ22447 promoted the reprogramming of NFs into CAFs by up-regulating IL-33 level in oral squamous cell carcinoma (Ding et al., 2018). LncRNA LINC00092 interactes with 6-phosphofructo-2-kinase/fructose-2,6-biphosphatase 2 (PFKFB2) to sustain the CAFs-like features of fibroblasts within tumor microenvironment and promote ovarian cancer metastasis (Zhao et al., 2017). The transfer of bioactive molecules from malignant cells to stromal cells via exosomes is also a key regulator of CAF differentiation. To date, at least two dozen miRNAs have been identified in various cancer types mediate CAF differentiation through exosomes (Shoucair et al., 2020). Some studies have confirmed that the expression of lncRNA-containing exosomes released by some tumor cells upregulates in the stroma and also participate in the activation of CAFs, but researches hitherto remain sparse. A recent study found that exosomal lncRNA PCAT-1 involved in tumor stroma remodeling in lung cancer. High expression of PCAT-1 can trigger the differentiation of fibroblasts into CAFs. Rather, PCAT-1 knockdown impaired CAF-mediated stromal activation and tumor growth *in vivo* (Domvri et al., 2020).

### Tumor Proliferation and Migration

Perhaps the best-known function of CAFs is to promote tumor progression. Microarray gene expression analysis of CAF and NF cell lines showed that differentially expressed genes were mainly regulated by the TGF-β signaling pathway. CAFs can promote EMT and tumor cell metastasis in a variety of ways through TGF-β-dependent mechanisms (Calon et al., 2014). TGF-β1, which is partially secreted by CAFs, is a critical medium for CAFs to induce EMT and metastasis of lung cancer cells, and an important medium for the interaction between stroma and cancer cells (Kalluri, 2016). It has been previously reported that CAF-derived TGF-β11 promotes EMT and invasion of bladder cancer cells through lncRNA-ZEB2NAT (Zhuang et al., 2015). In breast cancer, CAFs activate HOTAIR transcription by secreting TGF-β1, thereby promoting the metastasis activity tumor cells (Ren et al., 2018). Similarly, TGF-β1 secreted by CAFs leads to overexpression of lncRNA (TBILA) in NSCLC tissues, which can be induced by the classical TGF-β1 /Smad2/3 signaling pathway, cis-regulating HGAL (a TGF-β-induced gene), and activating the S100A7/JAB1 signaling pathway, thereby promoting the progress of NSCLC (Lu et al., 2018). As a tumor suppressor gene in NSCLC, lncRNA ANCR inhibits the migration and invasion of NSCLC cells by down-regulating TGF-β1 expression (Wang et al., 2018). Evidence shows that CAF-derived exosome lncRNAs also play an increased role in tumor proliferation. Exosome-lncRNA SNAI1 secreted by CAFs induces EMT in lung cancer cells. However, treatment of CAFs with exosome release inhibitor GW4869 significantly inhibited the induction of EMT in recipient cancer cells (You et al., 2019).

### Tumor Immunosuppression

In recent years, immunotherapy has become a hot topic in cancer treatment. Nevertheless, only a small percentage of cancer patients benefit from tumor immunotherapy, and a number of patients develop resistance to the therapy (Cristescu et al., 2018; Horvath et al., 2020). There is growing evidence that TME is one of the vital factors in the regulation of tumor immune response, not only tumor cells themselves. CAFs interact with a variety of immune cells, both innate immune cells (macrophages, neutrophils, dendritic cells, natural killer cells, and bone marrow cells) and adaptive immune cells (T and B lymphocytes), to modulate tumor immune response (i.e., immunosuppression) and promote tumor progression (Piersma et al., 2020). CAFs-mediated effects can directly increase the amount of inhibitory T lymphocytes and counteract effector T cell function. In addition, CAFs produce ECM components that form a physical barrier against immune cell infiltration (Mhaidly and Mechta-Grigoriou, 2021). LncRNAs can also act as tumor promoter genes and suppressor genes to regulate immune cell and immune response in TME (Xue et al., 2021). For example, lncRNA NKILA promotes tumor immune escape in the lung cancer microenvironment by regulating T cell sensitivity to activation-induced cell death (AICD) and increasing CTL infiltration (Huang et al., 2018). Exosomes contain membrane surface related antigens, immune stimulation and inhibitory factors, and biological active substances, which can participate in the immune response (Xu et al., 2020).

There is evidence that the interaction between CAFs and lncRNAs can also mediate immune responses. Domvri et al. found that PCAT-1 in the lung tumor microenvironment promotes a pre-metastatic niche formation by immunosuppressive miR-182/miR-217 signaling and p27/CDK6 regulation. PCAT-1-activated CAFs also enhanced the transformation of tumor-associated macrophages (TAMs) into a tumor-supporting M2 phenotype. This leads to an increase in infiltrating macrophages in the microenvironment, which then progresses to an immunosuppressive phenotype (Hegab et al., 2019; Domvri et al., 2020). Teng et al. (2019) showed lncRNA profiling in NSCLC. The lncRNAs differentially expressed between the paired CAFs and adjacent normal fibroblasts of the three patients were detected by gene microarray. Compared with NFs, we found 322 up-regulated and 444 down-regulated lncRNAs in CAFs. Bioinformatics analysis methods such as Gene ontology (GO) and Kyoto Encyclopedia of Genes and Genomes (KEGG) were used to analyze these differentially expressed lncRNAs, and it was found that these dysregulated lncRNAs were associated with immune function. These results suggest that CAF-specific lncRNAs mediate immune responses during the progression of lung cancer. However, there are relatively few studies on CAFs directly regulating lncRNAs to induce immunosuppression. Most of them are due to the interaction between various cytokines secreted by CAFs (such as TGF, IL-6, IL-8, etc.) and lncRNAs to exert immunosuppression (Ghafouri-FardAbak et al., 2021). For example, lncRNA LINC00301 significantly accumulated Tregs and inhibited CD8^+^ T cell infiltration in the nude mouse tumor microenvironment of NSCLC cell lines by targeting TGF-β (Sun et al., 2020). The mechanism of CAF regulating lncRNAs to further play immune escape needs to be further explored. This provides a promising target for further study of immune regulation in lung cancer.

### Therapeutic Resistance

Accumulating studies have found that tumor drug resistance is not only related to tumor cells, but also closely related to TME. TME-mediated resistance can be induced by chemokine and cytokine secreted by tumor cells and stroma. Mink et al. (2010) found that 24% of CAFs in tumor matrix were generated by EMT-derived tumor cells via green fluorescent protein-tagging experiment, and expressed epithelial membrane protein-1, a gefitinib resistance biomarker, to promote EGFR-TKI resistance. LncRNAs also play significant roles in the regulation of drug resistance. CAFs can directly or indirectly lead to abnormal expression of lncRNAs in tumor cells. This abnormality may alter the drug sensitivity of tumor cells by affecting downstream ceRNA. Zhang et al. (2017) found Midkine derived from CAFs increases the expression of lncRNA ANRIL in lung cancer cells, ovarian cancer cells, and oral squamous cell carcinoma cells through paracrine action, thereby promoting the upregulation of ABC family proteins MRP1 and ABCC2, and ultimately leading to the resistance of tumor cells to cisplatin. In contrast, silencing lncRNA ANRIL can overcome MK-induced cisplatin resistance via activating the caspase-3-dependent apoptotic pathway. Similarly, CAFs upregulate the expression of lncRNA HOTAIR to inactivate caspase-3/BCL-2 signaling pathway and promote cisplatin resistance (Sun and Chen, 2021).

In addition, it has been shown that CAFs affect the therapeutic resistance behavior of tumor cells through the release of exosomes containing lncRNA. CAFs can activate the β-catenin pathway by transferring exosome lncRNA H19, promoting chemotherapy resistance in colorectal cancer (Ren et al., 2018). Exosomes with high expression of H19 can also promoted gefitinib resistance of NSCLC cells (Lei et al., 2018). Based on the above studies, it is possible to explore whether H19-exosomes in NSCLC also derive from CAF. LncRNA KCNQ1OT1 is also packaged into exosomes and secreted by CAFs, thus inducing cancer cell proliferation, promoting the G1/S transformation of cancer cells, and increasing radiotherapy resistance (Mao, 2019). The development of potential drugs that can target lncRNA and CAFs may be a solution to improve treatment resistance.

## The Diagnostic, Prognostic and Therapeutic Value of Long Non-Coding RNAs in Tumor Microenvironment

As functional RNA molecules, lncRNAs and lncRNAs-enriched exosomes control crucial pathways in microenvironment and are expressed in specific ways at specific stages of progression. They have gradually become new biomarkers and therapeutic targets for cancer. We provide our perspective on the clinical use of lncRNAs as therapeutic targets or prognostic/predictive biomarkers, contributing to the development of precision therapy.

### Application of Long Non-Coding RNAs as Biomarkers

Previous studies have found that abnormal expression of lncRNAs can regulate the interaction between tumor cells and stromal cells, which can be used to predict tumor behavior and patient prognosis. The ability to capture tumor interstitial correlation signals may be of great significance. The lipid bilayer of exosomes provides a protective membrane that encapsulates and protects biologically active molecules. Therefore, exosomes exist stably in various body fluids such as blood, saliva, bronchoalveolar lavage fluid, and sputum. Due to their small size, exosomes are easier to penetrate into a variety of body fluids, mediating signal and substance exchange between tumor cells and CAFs. Exosomes can be used as promising diagnostic and prognostic value due to these unique characteristics (Chung et al., 2020). Precise liquid biopsies can be performed by non-invasive methods to obtain exosomes abundant in body fluids. It is a promising method to improve early diagnosis and cancer prognosis. Abnormal expression of exosomes from tumor cells and mesenchymal cells are also associated with different stages of cancer (Barile and Vassalli, 2017). Dynamic monitoring of differential expression of bioactive molecules (such as lncRNAs, miRNAs) in exosomes can reflect tumor development more accurately and timely. However, the reliability and stability of extraction technology are the premise of exosome research. However, due to their nanoscale volume, efficient purification of exosomes from body fluids is full of challenges. In addition, the high cost of the test may limit its widespread use and large prospective clinical trials must be conducted to provide evidence of its clinical utility. At present, these are still great challenges to its clinical application as biomarker.

### Potential of Targeted Long Non-Coding RNAs Therapy

The roles of CAFs in cancer development makes them potential therapeutic targets. Nevertheless, due to the lack of specific CAFs markers, it is difficult to accurately target CAFs without damaging normal tissues. In addition, CAFs have both protumorigenic and antitumorigenic effects. Ongoing attempts to treat CAFs still face a number of challenges. A few of receptor antagonists targeting CAFs-derived paracrine factors are being developed to prevent tumor-stromal interactions. Siltuximab, as an anti-IL-6 monoclonal antibody, was found to have strong tumor suppressive effects in lung cancer xenograft models treated with CAFs (Nikanjam et al., 2019).

According to recent studies, targeting lncRNAs in CAFs is also a promising direction of exploration. Although miRNAs in the tumor microenvironment have been extensively studied. The regulation of mRNAs is realized by targeting miRNAs, and then the transcription and post-transcription regulation of functional genes are realized. Because one miRNA can regulate multiple mRNAs, and one mRNA is regulated by different miRNAs. Compared to targeting miRNAs, lncRNAi is equivalent to simultaneously targeting multiple different miRNAs molecules, thus improving the efficiency of tumor growth inhibition. Several approaches can target lncRNAs, such as antisense oligonucleotides (ASOs), short hairpin RNAs (shRNAs), short interfering RNAs (siRNAs), small molecule antagonists, and nucleic acid aptamers (Chen et al., 2019a). The degradation of lncRNAs in CAFs by ASO interferes with their phenotypes and tumor-friendly functions. In a mouse xenograft model, MALAT1 ASO is a potential treatment targeting cancer-associated lncRNAs, which can effectively inhibit lung cancer spreading (Gutschner et al., 2013). As a large hydrophilic compound, delivery of ASOs to target organs *in vivo* is one of the biggest problems encountered in drug development (Levin, 2019). The efficacy of oligonucleotide drugs in locally administered diseases is significantly better than that in systemic diseases. Nusinersen, the most successful antisense oligonucleotide drug, is administered by intrathecal injection for spinal muscular atrophy (Chiriboga, 2017). As the stable structure gradually became the marker of lncRNA, the specific identification of lncRNA domain by small molecule drugs was used to regulate the phenotype. And compared with ASOs, small molecule drugs have higher tunability properties. With molecular docking and high-throughput screening, a small-molecule compound AC1Q3QWB was identified to interfere with the expression of lncRNA HOTAIR and increase the expression of tumor suppressor (Li et al., 2019). It is currently difficult that targeting lncRNAs in a scalable and replicable way with small, drug-like molecules. In addition, screening methods should be improved to identify drug-like lead molecules with appropriate pharmacological properties and identify RNA motifs with sufficient informative content (Warner et al., 2018).

Exosomes are effective carriers for communication of genetic material and other information between cells. Inhibiting the production and secretion of exosomes can be a potential therapeutic method. Several compounds have been identified as potential inhibitors of exosome production. The most common exosome inhibitor is a non-competitive inhibitor of membrane neutral sphingomyelinase (nSMase) inhibitor GW4869. According to the bilayer lipid structure of exosomes, exogenous SMase can induce exosomes formation. Accordingly, the use of GW4869 can inhibit lipid metabolism and reduce exosome production, thus interfering with tumor progression (Catalano and O’Driscoll, 2020). Some studies have found that GW4869 blocks lncRNA ASLNCS5088-enriched exosomes in M2 macrophages, and then inhibits the regulation of M2 macrophages on fibroblast activation (Chen et al., 2019b). Ras-related protein RAB27A belonging to the RAB GTPase superfamily is involved in protein transport and small GTPase mediated signal transduction as a membrane binding protein. It has been found to play an important role in the production and secretion of exosomes. Reducing the expression of RAB27A can effectively reduce the secretion of exosomes (Zhang Y. et al., 2020). Sulfisoxazole (SFX), an FDA-approved oral antibiotic, has recently been found to act as an inhibitor of exosomes by interfering with endothelin receptor A (ETA) in breast cancer (Im et al., 2019). Interestingly, the mechanisms by which these compounds block exosome secretion vary from each other. Further studies are needed to better understand the role and targets of exosome in intercellular communication. In addition, most exosome inhibitors cannot selectively identify exosomes that are involved in pathology, rather than those that perform necessary physiological roles currently. The study of targeted inhibition of exosome secretion still needs substantial efforts.

## Conclusion and Perspectives

Cancer-associated fibroblasts (CAFs) are key components in TME that support tumor growth, generate a physical barrier against drug and immune invasion, and help regulate malignant progression. Activation and function of CAFs are mainly controled by a large number of factors, among which lncRNAs act in indispensable roles. Through lncRNAs, CAFs regulate tumor cell proliferation, metabolism, immune escape and treatment resistance. Tumor cells and CAF-derived lncRNA-loaded exosomes are also extensively involved in this interaction. Therefore, it is widely accepted that therapies designed to block the interaction between tumor cells and CAFs can enhance the effectiveness of anti-cancer therapies. Although there are only a few reports of lncRNAs in CAFs. To sum up, the results provide new insights into the communication between tumor stroma and cancer cells, and demonstrate the therapeutic potential of targeting lncRNA mediated cross-talk between cancer and stromal cells in cancer therapy. At present, there are still many obstacles and challenges to carry out targeted work on CAFs. The design of novel therapies targeting the tumor stroma depends on a better understanding of TME’s interactions with cancer cells.
